# “There is never any rest, never enough time and too much to do”: a qualitative study of GP work intensity in an Irish context

**DOI:** 10.1186/s12875-026-03321-6

**Published:** 2026-04-18

**Authors:** Holly Rose Hanlon, Éidín Ní Shé, Andrew W. Murphy, John-Paul Byrne, Laura Cullen, Susan Smith, Mike O’Callaghan, Niamh Humphries

**Affiliations:** 1 RCSI University of Medicine and Health Sciences, Dublin, Ireland; 2 University of Galway, Galway, Ireland; 3 Irish College of General Practitioners, Dublin, Ireland; 4 Trinity College Dublin, Dublin, Ireland

**Keywords:** Primary care, Work intensity, Medical workforce, Health system research

## Abstract

**Background:**

Globally, general practice faces recruitment and retention challenges as many countries struggle to meet the rising demand for primary care. Ireland also faces increased demand for primary care as a result of its ageing population, increased incidence of chronic illness and greater patient complexity. Significantly more GPs will be needed to meet this increased demand. Further challenges facing Ireland’s GP workforce include the fact that one quarter of GPs in Ireland are aged over 60 years old and changing working patterns by GPS which have seen GPs reduce their patient-facing hours. Work intensity is one of the factors driving GPs to change their working patterns. This paper identifies the key sources of work intensity in general practice in Ireland and considers how to improve GPs’ experiences of work with a view to improving GP wellbeing and retention.

**Methods:**

The study used a qualitative method of remote ethnography, with 20 participant GPs. Each GP participated in two semi-structured interviews and an eight-week instant messaging conversation via Threema. Ethical approval was granted by the institutional ethics committee and data collection took place from October 2024 to July 2025.

**Results:**

Participant GPs described a high level of work intensity, describing a “relentless” pace, long hours, and little opportunity for breaks. Sources of work intensity included GPs’ expanded scope of care, increased patient complexity, patient demands and the administrative workload. Participant GPs highlighted practice-level strategies that might reduce work intensity, including protected administrative time, and longer appointment times for complex patients. The results of the study are discussed in the context of the individual, organisational, and system-level solutions that may be deployed to address work intensity in general practice.

**Conclusions:**

General practice in Ireland is under considerable pressure, with increased work intensity driven by multiple sources. In response targeted strategies are necessary to reduce intensity and improve GPs’ experiences of work. In order to protect GP wellbeing, improve GP retention and secure the future sustainability of the GP workforce, the causes of work intensity must be better understood and factored into GP workforce planning.

**Supplementary Information:**

The online version contains supplementary material available at 10.1186/s12875-026-03321-6.

## Introduction

### Background

General practice globally is experiencing challenges in relation to recruitment and retention [1–2]. Other issues facing general practice include ageing workforces which contribute to staff shortages [1–3], along with other drivers such as inadequate recruitment, increased health worker migration, failure to train enough doctors to meet demand, and issues with the working conditions in general practice as a result of the GP staffing crisis [2–6]. Like many countries, Ireland is struggling to meet the rising demand for primary care as an ageing population presents an increased incidence of chronic illness and greater patient complexity [7]. It is estimated that the general practitioner (GP) workforce will need to increase by 33% to 42% of current (at the time of the estimates) GP numbers [8, 9]. These estimates vary depending on assumptions regarding projections in population increases, changes to healthcare policy and delivery, and the specific time period for the estimate. The most recent projections from the Economic and Social Research Institute (ESRI) suggest that patient demand for GP appointments will increase by 23–30% by 2040, with a corresponding need for an increased number of GPs (equivalent to a 24–31% increase in the current GP workforce) [10]. The Department of Health “estimate that 1.1 recent graduates are needed to replace the working time of a retiring GP” ([11], p.8).

The GP workforce in Ireland is ageing; in 2022, one quarter of Ireland’s GPs were over the age of 60 [7]. This is of particular importance to workforce planning, as older GPs tend to work longer hours [12]. In the current GP workforce, almost one-third of GPs report working 30 h or less a week in general practice, indicating changing working practices that need to be understood and incorporated into workforce planning [13]. A recent qualitative paper by the authors [14] describes these changes to GPs’ working lives, and explains how GPs are opting to reduce their patient-facing work in response to high work intensity. GPs also describe reducing their overall working hours in order to improve their work-life balance [14]. This paper further develops/builds on this research by focusing on the sources of work intensity for GPs in Ireland. GP retention is of significant importance for the Irish healthcare system, and understanding how GPs are responding to high work intensity, including by reducing patient-facing work or reducing working hours, is vital.

### Work intensity

Work intensity has been found to impact individuals’ experiences of and decisions around work, as well as their mental health and wellbeing outcomes. Alongside volume of work, work intensity has been cited by UK-based GPs as a key driver of intentions to quit. One third of participants highlighted “unsustainable” workloads as the biggest problem facing general practice [15, 16]. More generally, work intensity has been associated with major depressive disorder and generalised anxiety disorder in workers [17].

This is notable, as GP wellbeing has generally been shown to be poor; in 2019 93.8% of UK GPs were suffering from a minor psychiatric disorder, and nearly three quarters (72.7%) from severe exhaustion [18]. Burnout is of particular concern; for example 60% of primary care workers surveyed in Spain in 2021 were experiencing emotional exhaustion [19]. A recent meta-analysis of burnout in GPs found wide-ranging estimates (6–33%) of aspects of burnout across multiple countries [20]. In general, the literature suggests that the mental health and wellbeing of GPs is at risk. While work intensity has been linked to negative mental health outcomes generally [15–17], studies specifically focusing on work intensity among GPs are lacking. It is notable that work intensity is relatively understudied in medical settings despite the fact that it is often mentioned in the context of burnout [21].

Work intensity is a construct involving multiple elements; Horner et al. [22] proposed a model of physician work intensity which includes; actual task demands (mental demand, physical demand, time demand), and the individual’s experience of *managing* those demands (effort, performance, and frustration). They noted that perceptions of work intensity can be influenced by multiple factors and levels; the patient (complexity, interactions), provider (the physician themselves, temperament, level of training), and practice levels (e.g., inefficient management, scheduling, staffing etc.). More broadly, work intensity has been conceptualised as “…having too much to do, in too little time, at too high a pace, with too few resources” ([23], p.97).

Horner et al. [24] found that existing and validated quantitative measures of work intensity were suitable for use in a medical setting. In one of the few qualitative studies of work intensity in physicians, Jacobson et al. [25] found that significant sources of work intensity for doctors (namely the work done outside of direct patient-facing care) were missed when using only quantitative tools. The elements of work intensity have also been found to vary in terms of importance across medical specialties; for example, one study which included primary care physicians found them to have the highest intensity for time demands when compared to physicians working in other disciplines [26].

Significant differences in work intensity have also been found according to complexity of patients [27]. Studies indirectly examining elements of work intensity have found that indicators of lower levels of intensity (ability to provide longer consultations, lower levels of GP stress) are associated with better patient experiences (ratings of care, experiences of accessibility and availability), illustrating the importance of reduced work intensity for patients and for healthcare delivery [28]. In a UK context, it has been suggested that the combination of GP shortages and increasing complexity of GP work is intensifying workload which in turn is contributing to increased rates of burnout and attrition among GPs [29].

These studies illustrate the need to qualitatively examine sources of work intensity from direct and indirect patient care. It is important to ensure that research identifies the sources of GP work intensity in order to identify and tailor specific solutions. In the case of general practice, it is also worth noting that in addition to everyday clinical duties, GP principals also have to run the business aspect of their practice, which is different to many other specialities.

### The present paper

GPs work long hours in Ireland; one study using real-time measurement of workload found that the average GP working day was 9.9 h and that one quarter of their work was undertaken outside of the hours of 9 − 5 [12]. This study also showed the relatively high burden of paperwork/administrative tasks [12]. This is higher than the working hours found for Ireland in other studies [30]. This is also at the higher end of the range of working hours (33.5–51.1 h per week) reported by GPs in 31 European countries, Canada, and New Zealand [30].

As workload and patient complexity in general practice in Ireland increases [7], so does work intensity. This has implications for GP s working lives and for GP wellbeing. An increased prioritisation of primary healthcare as part of the Sláintecare healthcare reforms is likely to further increase work intensity in general practice as it has meant that free GP care has been expanded to all children under the age of 8, and has increased the proportion of the population eligible for free GP care [7, 31]. Almost all (95%) GPs in Ireland now participate in the Chronic Disease Management (CDM) scheme which involves primary care management of certain chronic diseases, and 2,400 GPs are signed on to the free contraceptive scheme, which allows women aged 17–35 to see a GP for free for contraceptive visits [31].

This paper considers experiences of work intensity among GPs in Ireland. The paper aims to examine which sources and components of work intensity are particularly salient for GPs in order to identify how to improve their experiences of work and to protect GP wellbeing and GP retention. It will use qualitative methods to outline the sources of work intensity among a sample of 20 GPs, addressing gaps in the literature on GP work intensity noted above. The paper also extends recent work by the authors [14] by considering the aspects of work intensity which are driving changing working patterns by GPs.

## Methods

This paper presents data collected as part of the GP Retention Project, a qualitative study seeking to explore the work experiences of GPs in Ireland. The first qualitative paper from the project [14] was on changing GP work patterns, and it showed that GPs are changing the way they work due to work intensity; the present paper focuses in on work intensity. The study used a method of remote ethnography called Mobile Instant Messaging Ethnography (MIME) which was developed by co-authors NH, JPB and used in previous studies of hospital doctors and public health doctors [32–33]. Further detail on the method is available in previous papers [33–34]. For this study, the MIME method involved participants (see Table 1 below) engaging in two semi-structured interviews and an 8-week instant messaging -based conversation with a researcher via Threema, all of which were guided by a theme sheet. The researcher sent three questions per week to participant GPs, which prompted an instant messaging conversation with participant GPs about their experiences of work. Discussions focused on topics such as work intensity, working hours, interactions with patients and colleagues, emotions at work, workplace supports, etc. Participant GPs also completed two qualitative interviews with the researcher (HRH) via Zoom (average interview length 45 min), one before and one after the MIME conversation, the first to build rapport, and establish background and demographic information about participants and their working patterns, and the second to further discuss issues raised in the MIME conversation, and broader questions relating to their work, their future plans and reflections on participation. Both the interviews and MIME were guided by theme sheets developed specifically for the GP Retention Project (see Appendix 1) and were conducted by HRH from October 2024 to July 2025. Ethical approval for the study was granted by the RCSI research ethics committee in June 2024.

### Participants

Participant GPs were purposively sampled through social media, Irish College of GPs webinars and newsletters, and via snowball sampling, to ensure a varied sample of GPs. All 20 participant GPs completed Interview 1; 19 also completed the MIME conversation and 18 also completed Interview 2. Informed consent was obtained from all participants prior to commencement of data collection.

**Table 1:** GP retention project participants (*n* = 20)

GP Retention Project participants (*n* = 20)		
Gender	Male	7
	Female	13
Caring responsibilities	Yes	15
	No	4
Years since qualifying	< 5 years	3
	5–10 years	3
	11–20 years	7
	20 + years	7
Location	Rural	11
	Urban	9
Country of training	Ireland	17
	Other	3
Country of GP training	Ireland	14
	Other	6
GP practice type	Single GP practice	3
	Two GP practice	3
	Multi-GP practice	14

### Analysis

Interviews were conducted and recorded via Zoom and transcribed using MaxQDA transcription services. The final dataset consisted of 57 transcripts (38 interviews, 19 MIME). All transcripts were then de-identified and circulated to participants for approval before being uploaded to MaxQDA for analysis.

During the initial analysis phase, work intensity was a pervasive theme for participant GPs. The initial interview contained one question on workload, and the MIME theme sheet contained specific questions on intensity, but the data presented here comes from the entire dataset (both interviews and the MIME conversation) as issues relating to work intensity were frequently raised by participants in all aspects of the study. Data from all three sources were considered within the analysis. Initially, the entire dataset was coded deductively by HRH for “busyness/work intensity”; this code included anything relating to the volume, intensity, pressure, or stress of work; often termed “busyness” by participants. This code sought to capture all responses relating to work intensity or influencing factors. The coded data extracts formed an “intensity” dataset which was then inductively coded by HRH to consider the nature of work intensity and to understand which aspects of work intensity were most salient for GPs. The inductive coding was guided by Braun and Clarke’s thematic analysis [35–36]; an extensive list of codes was generated and refined throughout the coding process. After this process, the individual codes were reviewed and grouped into several themes, which were reviewed and further refined to the three key themes described below. Theme and data alignment was reviewed and discussed with co-authors. Verbatim quotations from the data are presented in italicised quotes.

## Results

The three major themes resulting from the qualitative analysis are: the sources of work intensity for GPs, the impact of work intensity on GP wellbeing, and how to address GP work intensity. Quotes from interviews are indicated by the inclusion of the participant and interview number following the quote, quotes from the Threema conversations are indicated by the inclusion of the participant number and “Threema conversation” following the quote.

### Theme 1: sources of work intensity for GPs

The first theme relates to a pervasive feeling among participants that the GP workload is unmanageable, both in terms of volume and intensity. Participants spoke of an unrelenting pace of work, driven by patient demand, complexity, and the accompanying paperwork burden, with limited opportunities for breaks throughout the working day.

“*I would routinely go home in the evening and realise I haven’t had lunch. I might have had coffee. I will have had one cup of coffee. Generally no lunch. And likely not peed for a very extended length of time. So it is*,* it’s hectic*,* you know.*” (Participant 19, Interview 1).

“*There is never any rest*,* never enough time and too much to do.*” (Participant 1, Threema conversation)

Participants also spoke of regularly working outside of core working hours.

“*on either Saturday or Sunday*,* I’ll be doing an awful lot of paperwork*,* catching up with everything that’s gone on over the week*,* trying to get on top of it for the coming week.*” (Participant 12, Interview 1)

One participant described their working weeks as sometimes “*completely unmanageable*” and described those weeks at work as being “*Busy. Just*,* just really*,* really*,* just really*,* really*,* really busy. . the list of things you need getting done. . keeps getting bigger and bigger and you’re never going to get through it.*” (Participant 1, Interview 1).

Another participant explained that their workload is “*impossible. It’s just impossible. You just cannot keep it done. It’s as simple as that. . And it does seem to be increasing all the time and there is very little being done to fix it. . it is really it is a very*,* very busy job*.” (Participant 5, Interview 1).

There were a number of subthemes to the unmanageable workload which contribute to the level of work intensity experienced by GPs; namely expanded scope of care and increased patient complexity, patient demand, and administrative and paperwork workload. These are described below.

### Expanded scope of care and increased patient complexity

Participants spoke about the elements of work intensity that directly related to patients and the expanded role for GPs; “*the scope of GP has increased significantly over the last few years*,* and I think that’s a really good thing*,* in theory*,* you know*,* like there’s a lot of things that GPs do now that they didn’t do ten years ago…complex menopause care in practice…contraceptive care*,* inserting Mirena coils and Implanons*,* joint injections… even in things like diabetes care…. So I think we’re doing a lot more than we used to. And that means we’re probably having much more patient contact than we used to*,* per patient.*” (Participant 13, Interview 1).

Patient complexity was considered to contribute to the workload, due to increased numbers of patients with chronic illness and multi-morbidity and also due to structural changes to GP care, such as the introduction of the CDM programme. The CDM programme entitles public patients with specific chronic conditions to government subsidised bi-annual reviews by their GP. While this was viewed positively by most GP participants, it was also considered to have reduced their capacity for acute appointments.

“*I suppose GP is probably becoming more and more chronic care. You know*,* obviously the CDM is great for that and all the things that go with that*,* along with the contraceptive schemes um*,* you know and the increase in the doctor visit cards for the kids and everybody else*,* have made it more chronic care. So I think people are finding it harder and harder to get the acute stuff looked after.*” (Participant 3, Interview 1).

Complex patients require longer appointment times than the standard 10–15-minute appointment, they tend to generate more paperwork and involve more complex pharmaceutical management.

“*A number of complex consultations meant I was running behind. I was able to catch up and still leave on time but I have a number of things to catch up on this evening*” (Participant 10, Threema conversation)

The general role of the patient cohort in determining patient complexity was apparent in the data. GPs working in areas of deprivation are seeing patients who are generally sicker, have higher rates of substance misuse, and require higher levels of advocacy to ensure they receive the care they need. This impacted on work intensity, as this GP who works in an urban deprived area, explained:

“*We have about one GP per thousand patients*,* which is quite high staffing ratios. But even with that*,* we’re very busy like our patients are not well*,* you know*,* so they need to be seen more often*” (Participant 4, Interview 1).

GPs highlighted how different patients and patient groups may require more than the standard patient appointment time (10–15 min). For instance patients who do not speak English can need interpreters which can double appointment times; while elderly patients can have high complexity and co-morbidities and may also require more time with their GP.

“*I mean*,* 15 minutes. . was good when*,* when it was all just acute care and maybe one*,* one problem. But*,* we do a lot of the chronic disease*,* particularly*,* our population would be very multi morbid and elderly. And yeah*,* I mean*,* even the standard appointments can be fairly lengthy now*,* and you’re trying to get a lot of the chronic disease stuff done as well*,* never mind just the extended*,* you know*,* mental health appointment or whatever it is.*” (Participant 15, Interview 1)

Finally, participants spoke of both a higher level of pathology in rural general practice, and a tendency for rural GPs to manage patients to a higher level, because of a reluctance among their patient cohort to go to hospital, often due to longer distances and/or difficulty with transport, etc.

### Patient demand

Patient demand – that is the number of patients seeking appointments – was noted frequently by participant GPs. They spoke the difficultly of accommodating acute patients for same-day appointments, and how there is frequently a need to ‘squeeze in’ extra patients. GPs spoke of working at maximum capacity, with little time and space to accommodate staff absences or unexpected emergencies. The pressure on GP practices to take on the care of additional public patients from retiring GPs was also adding to capacity issues.

“. *. it’s constantly at max capacity*,* there doesn’t seem to be … a busy season in winter and like a less busy season in summer. It’s*,* it’s just busy or busier*.” (Participant 13, Interview 1)

Participants described increased patient expectations and demands. Although new developments in healthcare were seen as positive, they frequently came with an increased level of demand from patients (e.g., weight loss injections) for GP care.

### Administrative and paperwork workload

Participants described a significant administrative workload; the sheer volume of paperwork and administrative tasks were considered a major contributor to work intensity for GPs.

“*with the volume of patients that we’re seeing*,* like I literally could spend a full working week doing just that stuff of the like updating medication changes… And then the letters that are things like refer to this*,* refer to that. Follow up on this or checking that something’s done. And that’s a full time job.*” (Participant 19, Interview 1)

Participants also described the the administrative workload that they felt was avoidable; for example having to re-issue medical certs or prescriptions from hospitals, or having to follow-up on hospital referrals, or discharge letters containing insufficient details.

“*okay*,* I referred them for their hip replacement. Why am I making my 10th phone call? (.) And*,* you know. . this is such time wasting. Yet if I don’t do it*,* someone isn’t going to get their hip done*,* like this is just not something that I should be personally bearing.*” (Participant 20, Interview 2).

This issue of work transfer from other services was commonly mentioned by participants. These were matters that could be dealt with by other healthcare services, but that needed to be managed by GP, perhaps as a result of waiting lists or the need for a GP referral to access specific services. Managing and responding to these situations involved a significant administrative workload.

“*We’re getting lots of*,* more and more pushback from our colleagues who are under pressure*,* too… You know*,* and then we’ll get back a letter. Due to my waiting list*,* which is nine months*,* I would recommend you do the following*,* you know*,* and we’ve to write back*,* going*,* dear doctor*,* I’m not*,* I’m a general practitioner. I’m not indemnified*,* trained nor interested in doing your job for you. And it causes a little bit of frisson between primary and secondary care*,* you know. But I’m not taking on secondary care burden.*” (Participant 11, Interview 1)

Finally, participants spoke of the role played by GPs in “administrating the social welfare system” (Participant 1, Interview 1), with GPs acting as gatekeepers to a range of social supports for instance needing to complete disability forms or forms for housing benefit on behalf of their patients. GPs regularly receive requests from government agencies asking for “*health information for somebody to be able to access social supports… And*,* you know*,* you want to do the best for people*,* but you can’t also just accept being snowed under by constant requests for form filling and paperwork either*.” (Participant 15, Interview 1). This aspect of the administrative workload further illustrates the wide role of the GP and the increasing societal demands now placed at the door of the GP.

The data above illustrates that the administrative workload borne by GPs is not a singular entity, but rather is coming from multiple sources, from the paperwork generated from increased numbers of patients, to that generated as a result of the transfer of work from other services to GPs.

### Theme 2: impact of work intensity on GP wellbeing

GPs described the negative impact of work intensity on their mental and physical health, discussing both the direct and indirect of work intensity. Work intensity directly affected them by increasing their stress levels and their risk of burnout. Work intensity also had an indirect impact on their physical health by reducing the amount of time available to maintain their physical health, i.e. having limited time to exercise or to manage their existing health issues.

“. . *I have health issues. And I have absolutely no time to do the things*,* like I should be going to… that’s managed mostly by email to the consultant because I don’t actually have any time to get there. And that’s not me being dramatic or precious. That’s just a simple fact. . I actually can’t believe I’ve kept it going for years. You know*,* like*,* it’s*,* but that’s not without cost*,* you know*,* it’s it’s really not. For me*,* it’s probably been health and sanity.*”

(Participant 19, Interview 1)

Participants also mentioned that the workload prevented them from doing recommended self-care activities; “. . *once you start*,* there’s little time for just stopping*,* emptying your head*,* all these beautiful consultation models and Roger and ABBA and all of that stuff that we learned about in college. No*,* I do not reset at the end of every consult*,* emotionally or otherwise*,* to prepare myself and empty my head for the next one. No*,* I do not.*” (Participant 18, Interview 1).

Work-life balance also suffers, with some GPs highlighting the impact of their workload on their ability to engage in activities outside of work, including family commitments.

“*I have three teenage boys. Like*,* at least they’re not small anymore. But like. Yeah. Like*,* you know*,* last night one of them was upset because I*,* he had a rugby match and I couldn’t go to the rugby match*,* you know what I mean? … And to be honest*,* when I get home in the evening*,* [researcher]*,* I don’t have the energy to talk to them.* (Participant 6, Interview 2)

Finally, GPs described experiencing isolation at work; they were too busy during the work day to interact much with colleagues, whether chatting or collaborating/having staff lunches/meetings etc., with many participants describing a work environment in which they saw no colleagues, only patients throughout their working day.

“*I don’t see anyone all day…yeah*,* you know*,* no one’s taking breaks like no one’s having lunch or having coffee*” (Participant 13, Interview 1)

“*Feeling bad as I was so busy I didn’t notice one of our receptionists was sick and had to leave early*” (Participant 10, Threema conversation)

The level of intensity also prevented them from seeking support from colleagues, either because they didn’t have the time to do so, or because they were aware that everyone was under pressure, and they didn’t want to add to colleagues’ burdens.

“*We have brief chats every day*,* but I suppose everyone is conscious that everyone is overworked and under pressure so sometimes you don’t want to be adding to that*” (Participant 6, Threema conversation)

### Theme 3: how to address GP work intensity

It is clear from the data that the volume and intensity of work is high for many participant GPs, but participants also mentioned a number of factors which can mitigate the level and/or impact of the workload, primarily at a practice level.

Participants spoke of GP practices where clear boundaries enabled GPs to take their lunch break and finish work on time; others where GPs benefited from protected administrative time.

“*Yes no problem there today. But I was locuming in a practice that only sees 10 patients per session which is a far more manageable workload… The difference between 20 and 24 patients is huge. It’s basically takes out on hour of clinical work which you can spend doing admin stuff*,* in addition to obviously generating less admin to begin with.*” (Participant 1, Threema conversation, when asked if they left work when expected to that day)

Participants noted that GP practices make other decisions which influence GP work intensity, e.g. how many patients to take on, how long appointment times are and how many patients are assigned per GP session. It was acknowledged that financial and capacity considerations come into play for GP practices in making these decisions.

*“we have 30 minute mental health consults*,* which I think is a huge*,* huge benefit. Because when you have someone in with you for that type of consult*,* it can be quite overwhelming. But as well as that*,* you actually do want to listen to them*,* rather than just going through a checklist of stuff. So there is that flexibility to kind of practice in a way that meets*,* you know*,* your clinical needs as well as the patient’s.*” (Participant 15, Interview 1).

One participant spoke about the extent to which GP work intensity and busyness can be addressed at practice level.

“*Everyone talks about how busy GP is*,* but. . that is something you’ve control over. It’s not like hospital where anyone can turn up to your A&E and you have to see them*,* in GP*,* you know it’s your business*,* so. . so it’s up to you when you draw the line… the partners can decide when that happens. And that decision*,* I suppose*,* is busyness versus income.*” (Participant 12, Interview 1)

To an extent, GPs’ experiences of work intensity were dependent on practice level decisions around patient numbers, scheduling, locum cover and the availability of protected administrative time. At a practice level, the decision to enact these types of practices can be constrained by financial considerations. They may also sometimes out of the hands of GP principals, for instance if additional public patients are assigned to a GP practice by the HSE, increasing patient list sizes or the availability of locums to cover holiday and sick leave.

## Discussion

### Overall picture of GPs’ work intensity

The data presented in this paper shows that the volume and intensity of work among GPs in Ireland is very high; GPs regularly work outside of standard working hours, and experience high work intensity from multiple sources. At its most basic level, the work intensity described by participants relates to the workload, the number of people needing care, and the GPs’ experience of working to deliver that care. Overall, responses suggest a qualitatively similar experience of intensity to that cited by GPs in the UK [15, 16]. The clear time pressures and intense pace of work reported by participants in this study also aligns with that found by Horner et al. in the United States [26].

GPs spoke of high work intensity in relation to; increased scope of care, increased patient complexity and an increased number of patients in need of care. They also spoke about the administrative workload. They felt that work intensity was influenced by (and influenced in turn) factors operating at multiple levels; patient, physician, and practice; in this way the structure of work intensity for GPs appears to broadly map onto that conceptualised by Horner et al. [22]. This study has illustrated that work intensity is a significant element of GPs’ experiences at work. The picture of work intensity described by GPs here maps generally on to existing quantitative measures of work intensity such as the NASA-Task Load Index; future research might involve distributing the short-form quantitative measures of work intensity to GPs on a national level to quantify the general level of work intensity for GPs across the country. However, the qualitative nature of this study has allowed for a more in-depth exploration of work intensity for GPs, identifying the sources of GP work intensity, including increased demand for healthcare.

The picture painted by participants in this study is one of GPs working at or beyond their personal capacity, working long hours and at weekends and rarely taking breaks or time off work (even for sick leave or annual leave). GPs described the impact of this intensity on their wellbeing and work-life balance, with participants reporting insufficient time to look after their physical or mental health, or to engage in family commitments. GPs reported feeling isolated and having minimal interaction with their colleagues during the working day due to work intensity and heavy workloads. This level of intensity must change if we are to retain GPs. Our findings suggests that GP work in Ireland in 2025 meets the criteria of an extreme job in that it features unpredictable work patterns and long working hours, a fast pace of work with tight deadlines, a high level of responsibility and the need for 24/7 availability ([37], p.653, [38]. This has implications for GP health, wellbeing, and interpersonal relationships [39]. Those participants who work as GP Principals must also balance the business management side of their jobs alongside with their clinical duties.

The findings outlined in Theme 2, correspond to experiences described in the context of GP burnout; the effect of work intensity on GPs’ ability to take care of themselves physically and mentally, work-life balance, and isolation and lack of support-seeking are similar to the experiences described by GPs suffering from burnout [40]. GPs experiencing burnout have cited factors such as high workload, unrelenting time pressures, and long working hours, which suggest that the intensity of their work may be contributing to their burnout [40]. Other drivers of burnout in primary care workers include increased administrative or office work which was also discussed by GP participants in this study [41]. While this study did not directly explore GP mental health, the findings indicate a need to monitor GP wellbeing given the increased work intensity described by participants.

### What can be done? Areas to target

Particular areas were highlighted as contributing to the volume and intensity of work, some of which are more or less amenable to intervention. The administrative workload was repeatedly highlighted as an inefficient use of (and huge drain on) GPs’ time. Participants felt that the administrative work often involved duplication of work, or work that non-GPs could do, e.g. applying for social welfare benefits for patients. This is echoed in other studies which highlighted the “hidden workload” of non-patient-facing work [42] and the contribution of administrative work to pressure at work [15]. As illustrated above, the paperwork and administrative workload comes from multiple sources; an increased volume of tasks from direct patient demand, work transfer from other areas of the health system, increased paperwork as a result of the CDM programme etc., and each source will require targeted solutions. One potential general solution suggested elsewhere was to shift some of these administrative tasks to other allied healthcare professionals and administrative staff however, many of these tasks still require clinical supervision and task shifting (while not specifically for administrative tasks) has proved controversial in the UK [43]. Technological advances may provide other solutions; one participant (Participant 14, MIME) noted that their practice was trialling an AI dictation software to transcribe clinical notes; such software developments may assist GPs to deal with the administrative workload, along with strategies to reduce this workload in the first instance.

With regard to the transfer of work from other services to GPs, the Irish College of GPs recently published guidance for members to help address “inappropriate transfer of responsibilities from other healthcare providers to general practice” ([44], p2.). The guidance highlighted the potential for patient safety concerns and gaps in continuity of care when GPs are asked to manage and organise the follow-up to care initiated in other services and they support GPs who decline such requests. This statement illustrates the extent of this issue for GPs in Ireland, and suggests that some of this workload could be removed by ensuring that the responsibility of care remains with the services that began that care.

The expanding scope of care for GPs and the increased proportion of ageing and complex patients, increasing use of online clinics (often offered by health insurers) and walk-in clinics (which do not require an appointment) as a means to access acute care indicate that the nature of GP work is changing. The findings of this study indicate that these changes are accompanied by increasing intensity. The endurance of the full-time, 10 session work week for GPs (the Irish College of GPs states that one full work day contains two sessions [45]) may be less sustainable as a result. The reduction in patient-facing hours by many GPs supports this [14] and is an example of an individual solution to the health system problem of high work intensity [38]. Other individual responses to increased work intensity include working longer hours, working through lunch, and not taking leave were highlighted by participants. All have clear implications for the work-life balance and wellbeing of GPs and are not sustainable in the long-term. With the high intensity of GP work outlined in this paper, and the resulting changes to GP work patterns outlined by Humphries et al. [14], the ratio of 1.1 new GPs needed to replace every 1 retiring GP recently estimated by the Department of Health [11] seems a conservative estimate that may prove to be out of touch with the realities of GP work on the ground. If newly qualified GP graduates opt for portfolio careers [46] with less-than-full-time hours in patient-facing GP practice, then more of these graduates will be needed to replace retiring GPs who currently work full-time (or greater than full time).

There are also solutions to be found at an organisational level. While the underlying level of work intensity was high across the board, it could be mitigated to some extent by decisions made by GP practices to manage the demand. These practice-level strategies include closing patient lists (choosing not to take on any more patients), reducing the number of patients seen per day, and allowing longer appointments to address complex patients. However, this may in turn impact on demand by reducing the number of appointments available. It may also reduce practice income by reducing the number of patients seen. Similarly, the provision of administrative sessions were seen as a means of reducing time spent out of hours on administration. This kind of practice-level management of work intensity is something that is clearly already happening locally. Some GP practices in Ireland have closed their patient lists, with 17% of GPs unable to accept any new patient [47], but this reduces financial income for practices. It was noted by some participants that taking on new patients (to improve GP access for a local community) needs to be balanced with ensuring enough practice resources are available for patients already registered.

Finally, system-level solutions are needed in the form of need/demand-based resourcing in primary care. GP workforce planning should adapt to accommodate the increasing intensity of GP work, and the subsequent changes in GP work patterns [14]. It should also address differences in health needs locally, with Scottish data suggesting that GPs in the most deprived areas consult with 20% more patients than those in the most affluent areas [48]. Rural GPs also have unique challenges and needs and are under significant pressure; there are fewer GPs per population in rural areas, and these GPs are often operating as sole practitioners in populations which are older and which have greater health needs [7].

There is a need to plan for a sufficient GP workforce to enable practices to limit patient numbers and allow protected administrative time in order to safeguard GP wellbeing while ensuring continued GP access to patients. Supporting and planning for different ways of working may be one system-level strategy for ensuring a sustainable GP workforce into the future. Of course, building this slack into the system to allow for more sustainable levels of work intensity, whether via reduced patient numbers or patient-facing hours, will also result in the need for a greater number of GPs being trained and added to current workforce projections. Ultimately there is a need for more GPs in the system to deal with the main underlying driver of intensity, which is increased patient demand. There appears to be a conflict between the needs of individual GPs, the needs of GP practices, and the needs of the wider health system; clearly, GP practices limiting their patient numbers etc. will have implications for workforce requirements into the future, much as individual GPs changing their working practices to cope with intensity will.

Future research should examine the potential organisational and system-level solutions that might help to mitigate GP work intensity.

### Limitations

The study findings may by limited in their transferability due to the small, qualitative sample, though the sample size had national representation and the themes arising in the study reflected those identified in the international literature. There was also approximately a 2:1 ratio of female to male participants, which is an overrepresentation of female GPs relative to the actual proportion – 52% - of female GPs in the workforce [49]. Finally, participant age and ethnicity were not collected for this study, which limits comparison of our sample population to the wider GP population demographics.

## Conclusions

General practice in Ireland is under considerable pressure, driven by an expanding scope of care, increased patient complexity, high patient demand, and an increasing administrative and paperwork burden. GP workforce planning must account for the increased work intensity for GPs, and the accompanying changes to GP work patterns, in order to protect GP wellbeing and the sustainability of the GP workforce into the future.

## Supplementary Information

Supplementary Material 1.

## Data Availability

The dataset generated and analysed in the GP Retention Project are not publicly available due to privacy and confidentiality concerns. Reasonable requests for access will be considered by the corresponding author in collaboration with the institutional ethics committee.

## Abbreviations

GP: General practitioner
ESRI: Economic and Social Research Institute
CDM: Chronic Disease Management
