# Supplementary material for: “There is never any rest, never enough time and too much to do”: a qualitative study of GP work intensity in an Irish context
Source: BMC Prim Care. 2026 Apr 18;27:212. doi: 10.1186/s12875-026-03321-6 (PMC13220531; doi:10.1186/s12875-026-03321-6)
Supplement: Supplementary file 1 — Supplementary Material 1. [file 12875_2026_3321_MOESM1_ESM.docx]

Appendix 1 – Theme sheets

**GP-R Project**

**Interview 1 Theme Sheet**

**Background Information**

Did you complete your medical degree in Ireland, or elsewhere?

Did you train as a GP in Ireland?

When/where did you begin working as a GP? Do you have any caring roles and responsibilities outside work? (Children, parents, partner?)

Confirm gender.

What type of practice do you work in (single handed/group (if a group, what size? 1-2 people, 3-5, 5-10?)/corporately-owned)

Are you a partner in a practice?

Is the GP practice that you work in at the moment in a city, town or rural area?

**Working as a GP**

Within a typical working week, how many sessions do you work?

What other work do you do alongside your GP sessions?

Could you describe a typical working day for you?

What three words would you use to describe your work?

- (Interviewer to invite to expand on each of these one at a time after this)

How manageable is your workload at the moment?

Could you describe a typical working week for you? How would you describe your working week in terms of working hours/work life balance?

How well supported do you feel at work? [prompts: What does support at work look like? Who supports you?]

*The importance of GPs in coordinating specialist care for their patients came up in our discussions with patients. Could you tell me about the process of referring patients for specialist care? [prompts: How do you access it, are there any strategies that you use to speed up the process, what makes it easier or harder to access?]*

*Patients also mentioned that they greatly value the continuity of care and relationship they can have with their GP. Is this something that is also important to you?*

- *How does that work in practice (or does it work in practice/is that always possible to achieve in practice)?*

*How is your working day planned in terms of patient appointments? [prompt: Do patients with chronic conditions typically get assigned to the same GP? How do admin assign patient appointments to you?]*

**Reflecting on being a GP**

Have you noticed any changes to your work in the past 5 years?

Have those changes affected how you **feel** about your work?

**GP-R Project**

**Threema Message Prompt Theme Sheet**

**Welcome to the GP-R Threema conversation**. Thanks again for taking part in this study, we really appreciate your help. As we explained when we last spoke, we will send you 3 messages each week over the next 8 weeks (a total of 24 questions), asking about work-related issues. Please respond whenever you find time.

Please respond whenever suits you

Respond to questions whenever you find the time e.g. straightaway, at the end of the day, in evening, at the end of the week, or catch up the following week.

You are very welcome - and encouraged - to engage with us outside of the three questions per week should you want to/should something pertinent arise.

We will have a half-way check-in to ensure you’re happy to continue to participate.

- Feel free to reply to the messages with text, or by sending photographs, images, videos, documents, or audio or video recordings, whichever suits you best.
- We will remove any private information on these messages (we will mask, delete, remove or blur them).
- If there’s anything extra you would like to add, at any stage, just send us a message on this number XXX XXXXXXXX.

**Week 1: This week, we’ll be asking about your workload and your work-life balance.**

1. How was your workload today - did you have enough time to get everything done?
2. How would you describe your current work/life balance? [e.g. is it optimal, less than optimal?]
3. What was the most challenging part of your workday today?

**Week 2: This week, we will be asking about the complexity/intensity of your work.**

1. What was the complexity of your work like today? (e.g., did you deal with many complex cases, chronic illness, comorbidities etc.?)
2. How would you describe the balance between more complex cases and more “routine” cases in your working day today?
3. Did your work day today involve a significant amount of liaising with secondary care?

**Week 3: This week, we will be asking about your working hours.**

1. What were your working hours like today? (e.g. did you leave work when you expected to?)
2. Are you clear about what the ‘normal’ role of a GP is? (has it changed in recent years?)
3. Did you have to do work beyond your normal role today? (if you did, could you give examples of the type of ‘extra’ work you took on)

**Week 4: This week, we will be asking questions about staffing levels in your practice.**

1. Do you feel that there were enough GPs and other staff working in your practice to deliver the level of care required?
2. Is locum recruitment an issue for your practice?
3. Is staff turnover an issue for your practice?

**Midway Check in:** We greatly appreciate your input to the GP-R project so far.. We just wanted to check-in to ensure you’re happy to continue your participation?

**Week 5: This week, we will ask you about your in-practice interactions and workplace culture.**

1. How was your interaction with patients today? (giving specific examples if possible)
2. How was your collaboration with other people at work today? (Any examples come to mind?)
3. What was the most uplifting part of your workday today?

**Week 6: This week, we will ask you about emotions at work**

1. Could you tell me about the emotions you’ve felt at work this week?
2. How has your work impacted on your emotional wellbeing this week?
3. Does managing patients who are awaiting access to specialist care have an impact on your emotional wellbeing? If yes, could you describe this impact?

**Week 7: This week, we will ask you about the intensity of your work.**

1. What was the intensity of your work like today? (e.g. did you have time to take regular breaks during your working day, did you get everything done within your working hours?)
2. How would you describe the balance between administrative and direct patient care in your working day today?
3. What type of administrative work did you do today?

**Week 8: This week, we would like to ask you how well supported you feel at work.**

1. This week at work, have you felt well-supported and what has made you feel well supported?
2. Who provides you with work-related support? (e.g. is it colleagues, family, friends, etc.?)
3. What is on your mind this week when it comes to work? (Is there anything we haven’t asked you about that we should ask you about?)

**Thank you**

We greatly appreciate your input to the GP-R project and appreciate the insights that you’ve shared with us over the past few weeks. This message marks the end of our Threema conversation (from our end).

We will be in touch with you again shortly to arrange the next steps, but in the meantime if there are any further reflections that you’d like to share with us, please feel free to send them on.

Thank you again from the GP-R team.

**GP-R Project**

**Theme Sheet (Interview 2)**

**Introduction**

Thank you for taking part in WhatsApp conversations with us over the past 8 weeks. We’ll now ask you a few questions which will reflect back on the WhatsApp conversations and your earlier interview with us.

So**, reflecting back on your participation in the study** over the past few weeks, how did it feel to reflect on and discuss your work life in this way? Is this something you tend to do normally, or was this the first time you’d really taken time to reflect?

**Issues arising in the WhatsApp Conversations**

I noticed as we were chatting over the past few weeks that you mentioned (prominent theme that arose in conversation) a lot, it came up across a lot of the different questions we asked you. Is that a particular issue for you at work? [Repeat as needed]

One thing that struck me as we were chatting over WhatsApp over the last few weeks is that (expected theme not arising) did not come up in our conversations. Why do you think that is? [Repeat as needed]

**You at work**

Is there anything you feel that we ***should*** have asked you about work, but didn’t?

Do you feel that you have the support you need to do your job to the best of your ability?

Who do you receive this support from (GP colleagues, practice colleagues (not GPs), IGCP, union)?

**Your Work**

How would you describe the GP practice where you work?

What is the best thing about your GP practice

If you could change one thing about your GP practice, what would it be?

**Being a GP in 2024**

If you were to give advice to your younger self about being a GP (knowing what you know now), what would it be?

Is there anything about working as a GP that keeps you up at night? (prompt, that you find stressful?)

What are your future plans? And have they changed at all over the past five years?
